# Differential impact of the expression of the androgen receptor by age in estrogen receptor–positive breast cancer

**DOI:** 10.1002/cam4.138

**Published:** 2013-09-30

**Authors:** Eriko Tokunaga, Yuichi Hisamatsu, Kenji Taketani, Nami Yamashita, Sayuri Akiyoshi, Satoko Okada, Kimihiro Tanaka, Hiroshi Saeki, Eiji Oki, Shinichi Aishima, Yoshinao Oda, Masaru Morita, Yoshihiko Maehara

**Affiliations:** 1 Department of Surgery and Science, Graduate School of Medical Sciences, Kyushu UniversityFukuoka, Japan; 2 Department of Comprehensive Clinical Oncology, Graduate School of Medical Sciences, Kyushu UniversityFukuoka, Japan; 3 Department of Anatomic Pathology, Pathological Sciences, Graduate School of Medical Sciences, Kyushu UniversityFukuoka, Japan

**Keywords:** Androgen receptor, breast cancer, estrogen receptor, phenotype, postmenopausal

## Abstract

We evaluated the expression of the androgen receptor (AR) to determine its significance in breast cancer. AR expression levels were analyzed in 250 invasive breast cancers by immunohistochemistry and any association with the clinicopathological features was evaluated. AR expression was higher in estrogen receptor (ER)-positive cases than in ER-negative cases (*P* < 0.0001). AR expression was associated with ER level, and it increased with age in ER-positive cases. The cut-off value was determined to be 75% (*Cancer Res*. 2009;69:6131–6140), and AR expression was considered to be high in 155 (62%) cases. High AR expression significantly correlated with lower nuclear grade (*P* < 0.0001), ER and progesterone receptor (PR) positivity (*P* < 0.0001 and *P* = 0.0022), HER2 negativity (*P* = 0.0113), lower Ki67 index (*P* < 0.0001) and a longer disease-free survival (DFS) and distant metastasis-free survival (DMFS) (*P* = 0.0003 and 0.0107). This association between a high AR expression and a good DFS and DMFS was significant for ER-positive tumors (*P* < 0.0001 and *P* = 0.0018); however, no association existed between AR expression and prognosis for ER-negative tumors. In patients ≤51 years old, a high AR expression level significantly correlated with a better prognosis, but this was not significant in patients who were 50 or younger. Multivariate Cox hazard analyses revealed AR expression to be independently associated with a good prognosis in overall patients (HR 0.46, *P* = 0.0052) and in the ER-positive cohort (HR 0.34, *P* = 0.0009). AR expression is associated with a less aggressive phenotype and a good prognosis in patients with ER-positive breast cancer. This is considered to be a specific phenomenon for postmenopausal breast cancer patients.

## Introduction

The androgen receptor (AR) is a member of the steroid receptor subfamily. There is emerging evidence that the androgen signaling pathway may also play a critical role in normal and malignant breast tissue [1]. The AR is the most prevalent sex steroid receptor in malignant breast tumors, and is expressed in up to 90% of primary tumors and 75% of metastasis [2]. Previous studies revealed the expression of the AR to positively correlate with the estrogen receptor α (ERα) and progesterone receptor (PR) expression, low-grade, low proliferation activity, and advanced differentiation [3–11]. Several studies have demonstrated that the positivity for AR expression is associated with a better prognosis, especially in patients with ERα-positive breast cancers [1, 7–10]. In addition, the higher expression levels of the AR were associated with a better prognosis. This suggests that the AR may have a tumor-suppressive effect in breast cancer cells [1, 7, 9, 10]. Peters and colleagues showed that the AR is a direct repressor of ERα signaling in breast cancer cells [1].

Thus, the expression of AR is considered to be a good prognostic marker for ERα-positive breast cancer; however, there were some problems in previous studies. For example, the methods used to determine the positivity of AR expression were different among the studies [3–11]. Moreover, even when the expression was assessed by immunohistochemistry (IHC), the cut-off value of the expression of the AR differed among the studies [1, 7–11].

The role of androgens in the development and progression of breast cancer has not yet been fully elucidated. The mechanism underlying estrogen production dramatically changes before and after menopause. In postmenopausal females, adipose tissue is the primary source of endogenous estrogen production, rather than the ovary. After menopause, androgens (which derive mainly from the adrenal gland) become an important source of estrogens [12]. The ratio of circulating estrogens and androgens changes drastically after menopause [13]. Thus, the role of androgens or the AR in breast cancer might differ by age or menopausal status. Although previous studies have examined the effects of androgen based on menopausal status, the relationship between the role of the AR and the age of breast cancer patients has not been reported previously. The present study investigated the expression of the AR by IHC and the relationship between AR expression and clinicopathological factors in primary invasive breast cancer. In addition, we evaluated the clinical significance of AR expression by age and ER status. In agreement with previous studies, AR expression correlated with less aggressive features in ER-positive breast cancer. We found that its expression is significantly associated with a less aggressive phenotype and a better prognosis in females aged 51 or older, but not significant in those who were 50 or younger, with ER-positive breast cancer.

## Materials and Methods

### Patient information

Four hundred sixty-six primary breast cancer patients underwent surgery in the Department of Surgery and Science, Kyushu University Hospital, between 1997 and 2007. Among these patients, eight had stage IV disease, 29 cases were non-invasive ductal carcinoma and 34 cases were a special type of invasive cancer. Among the remaining invasive ductal carcinoma cases, a total of 250 cases for which archival tissue samples were available for an immunohistochemical analysis were included in this study. Written informed consent was obtained from all patients before collecting tissue samples. AJCC/UICC TNM Classification and Stage groupings were used.

### Immunohistochemistry to detect AR expression

The expression of the AR was analyzed by IHC. Formalin-fixed, paraffin-embedded tissue specimens were used, and the sections were deparaffinized with xylene and rehydrated. AR expression was analyzed as follows: The sections were first treated with the target retrieval solution (pH 9.0) (Dako, Glostrup, Denmark) in a microwave at 99°C for 30 min for antigen retrieval. The slides were then treated for 30 min with 3% H_2_O_2_ in methanol to block the endogenous peroxidase activity. Nonspecific antibody binding was blocked by incubating the sections with normal goat serum (Dako) for 10 min. The slides were then incubated with mouse monoclonal AR antibodies (AR441, diluted 1:50; Dako) [7, 9–11, 14] overnight at 4°C, and the samples were subsequently labeled with the Envision Detection System (DAB; Dako) for 1 h at room temperature. The sections were then developed with 3,3′-diaminobenzidine tetrahydrochloride (Dako) and counterstained with 10% Mayer's hematoxylin, dehydrated, and mounted. The expression of the AR was scored as the percentage of nuclear staining in a maximum of 1000 cells per sample.

### Evaluation of ER, PR, HER2, and Ki67 expression

The ER, PR, and HER2 status was evaluated as described previously [15]. The ER and PR were considered to be positive if ≥1% of the nuclei of the tumor were stained by IHC [16–18]. Tumors were considered to be HER2-positive if they were scored as either 3+ on IHC or as 2+ on IHC with HER2 amplification (ratio > 2.0) detected by fluorescence in situ hybridization [16]. Ki67 was evaluated as described previously [19].

### Statistical analyses

All molecular and IHC analyses were performed by investigators blinded to the clinical data. The statistical analyses were done using the JMP software package, version 9.0.2 (SAS Institute Inc., Cary, NC). The associations between AR expression and clinicopathological characteristics were assessed using χ^2^ tests. Survival curves were plotted using the Kaplan–Meier method and the log-rank test was used to determine the associations between individual variables and survival. The survival data were evaluated using a multivariate Cox proportional hazards model. Differences were considered to be significant at *P* < 0.05.

## Results

### Expression of the AR detected by immunohistochemistry

**Figure 1:**
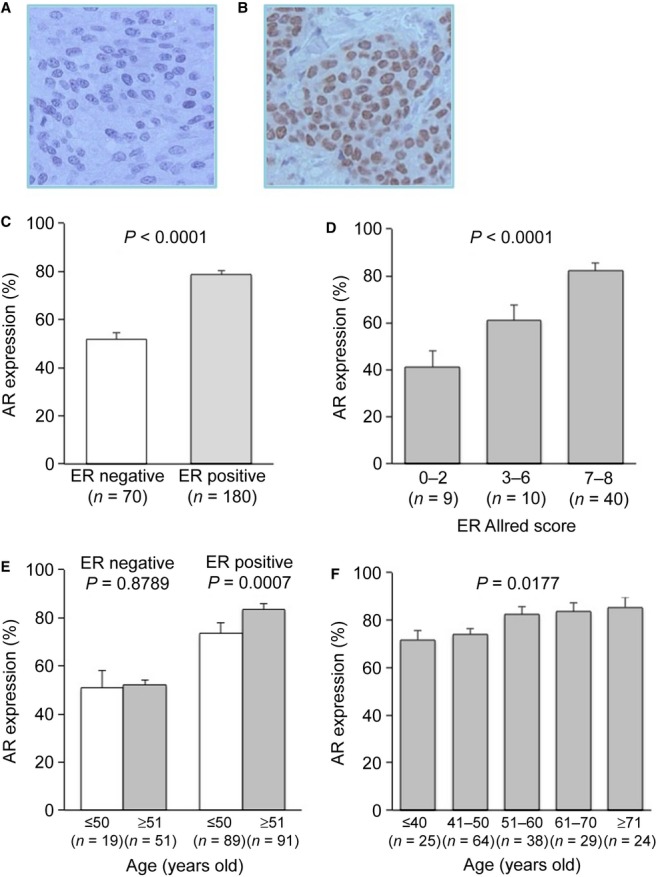
Results of the immunohistochemical analysis of androgen receptor (AR) expression levels in breast cancers. Representative images showing the negative (A) and positive (B) expressions of AR as evaluated by immunohistochemistry. Original magnification, 400×. (C) AR expressions were higher in estrogen receptor (ER)-positive breast cancer. (D) AR expression levels were associated with ER expression levels. (E) The relationship between AR expression and age in the ER-negative and ER-positive breast cancers. There were no significant differences in AR expression levels by age in ER-negative cases; however, AR expression was significantly higher in ER-positive patients who were 51 years old or older. (F) AR expression increased with age in the ER-positive cases.

AR expression levels were analyzed by IHC. AR immunoreactivity was observed in the nuclei of tumor cells. Figures 1A and B show representative photographs of low and high expressions of the AR. The mean percent of AR expression was 71.1, the median percent was 83.8, and the range of AR expression was 0–99%. AR expression was higher in ER-positive cases compared with ER-negative cases (mean 78.6 ± 1.5% vs. 51.8 ± 3.9%, *P* < 0.0001; Fig. 1C), although the range of immunostaining in both groups was identical (0–99%). The Allred score of ER expression was available for 59 patients. In terms of the relationships between ER Allred score and AR expression, AR expression was higher when the ER Allred score was higher (*P* < 0.0001; Fig. 1D).

In addition, we found an intriguing phenomenon wherein AR expression was different by age only in ER-positive cases. The mean age at natural menopause in Japanese females is 50 years [20]. Therefore, we divided all of the cases into two groups by age: ≤50 and ≥51 years old. AR expression was similar in ER-negative cases in both age groups; however, it was significantly higher in the older group of patients with ER-positive cancer (*P* = 0.0007; Fig. 1E). In addition, AR expression increased with age in the ER-positive cases (*P* = 0.0177; Fig. 1F).

### Associations between AR expressions and clinicopathological characteristics

The cut-off values used to classify AR expression were different among previous studies; however, the mean and median expression percentage and range detected in the current study are similar to those described in Peters' study [1]. Thus, in order to evaluate the associations between AR expression and clinicopathological factors and prognosis, we determined the cut-off value to be 75% according to their report [1]. AR expression was thus considered to be high in 155 (62%) and low in 95 (38%) cases. Table 1 shows the associations between the expression of the AR and the clinicopathological characteristics. High expression of the AR was significantly correlated with lower nuclear grade (*P* < 0.0001), ER and PR positivity (*P* < 0.0001 and *P* = 0.0022), HER2 negativity (*P* = 0.0113), and lower Ki67 index (*P* < 0.0001). Most of the tumors with high AR expression were hormone receptor-positive and HER2-negative cases (*P* < 0.0001; Table 1). There was no significant difference between age and AR expression in all cases. However, the frequency of high AR expression was significantly higher in females ≥51 years old in the ER-positive cases (*P* = 0.0088; Table 1).

**Table 1:** Associations between androgen receptor (AR) expression and clinicopathological characteristics

	AR		
		
Factors	Low (*n*=95)	High (*n*=155)	*P*-value
Age			
≤50	45 (47.4)	63 (40.6)	0.2981
>50	50 (52.6)	92 (59.4)	
ER positive only			
≤50	32 (65.3)	57 (43.5)	0.0088
>50	17 (34.7)	74 (56.5)	
Lymph node metastasis			
Negative	54 (56.8)	90 (58.1)	0.8495
Positive	41 (43.2)	65 (41.9)	
Tumor size			
T1	39 (30.5)	80 (51.6)	0.1376
T2	49 (51.6)	60 (38.7)	
T3	7 (7.4)	15 (9.7)	
Nuclear grade			
1	29 (30.5)	81 (52.3)	<0.0001
2	19 (20.0)	40 (25.8)	
3	47 (49.5)	34 (21.9)	
ER			
Negative	46 (48.4)	24 (15.5)	<0.0001
Positive	49 (51.6)	131 (84.5)	
PR			
Negative	55 (57.9)	59 (38.1)	0.0022
Positive	40 (42.1)	96 (61.9)	
HER2			
Negative	69 (72.6)	133 (85.8)	0.0113
Positive	26 (27.4)	22 (14.2)	
Subtype			
HR+/HER2−	47 (49.5)	123 (79.4)	<0.0001
HR+/HER2+	7 (7.4)	12 (7.7)	
HER2	19 (20.0)	10 (6.5)	
Triple negative	22 (23.2)	10 (6.5)	
Ki67 index (%) (mean±SE)	23.7±1.5	14.2±1.2	<0.0001
Adjuvant therapy in ER-positive cases			
None	2 (4.1)	13 (9.9)	0.1413
HT only	18 (36.7)	60 (45.8)	
CT only	15 (30.6)	22 (16.8)	
HT+CT	14 (28.6)	36 (27.5)	

ER, estrogen receptor; PR, progesterone receptor; HT, hormone therapy; CT, chemotherapy.

### Association between AR expression and prognosis

**Figure 2:**
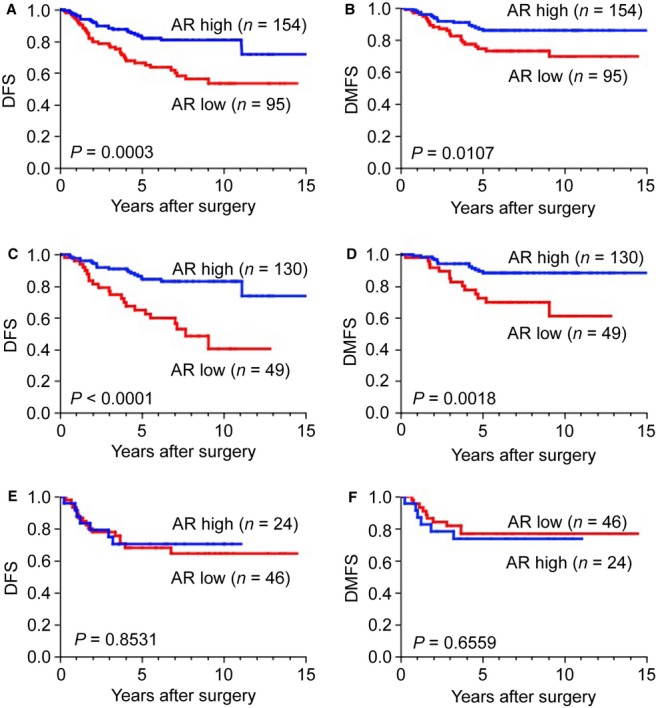
Relationship between disease-free survival (DFS) or distant metastasis-free survival (DMFS) and androgen receptor (AR) expression. (A and B) A high AR expression level was significantly associated with a longer DFS (A) and DMFS (B) in all cases. (C and D) A high AR expression level was correlated with a better prognosis in ER-positive cases in terms of both DFS (C) and DMFS (D). (E and F) No difference was observed in relation to AR expression in ER-negative cases in terms of both DFS (E) and DMFS (F).

The association between AR expression and prognosis was also evaluated. The median follow-up period was 6.6 years (range, 0.5–16.3 years). A high level of AR expression was associated with a significantly longer disease-free survival (DFS) and distant metastasis-free survival (DMFS) than a low AR expression (*P* = 0.0003 and 0.0107; Fig. 2A and B). This association between high AR expression and a good prognosis was significant in ER-positive tumors in terms of both DFS and DMFS (*P* < 0.0001 and *P* = 0.0018; Fig. 2C and D); however, there was no association between AR expression and DFS and DMFS in patients with ER-negative tumors (Fig. 2E and F). Regarding the adjuvant therapies prescribed in ER-positive cases, there were no significant differences between the AR-high and AR-low groups (Table 1).

Univariate and multivariate analyses were performed to assess the differences in DFS between the groups (Table 2). In addition to tumor size, lymph node metastasis, nuclear grade, HER2 status, and Ki67 index, AR expression was found to be significantly associated with DFS by univariate analysis. The multivariate Cox hazard analyses revealed that AR expression, as well as lymph node metastasis and nuclear grade, was independently associated with a good prognosis in the overall study population (high vs. low AR expression: HR, 0.46; 95% confidence interval, 0.26–0.79, *P* = 0.0052) and the ER-positive cohort of patients (high vs. low AR expression: HR, 0.34; 95% confidence interval, 0.18–0.64, *P* = 0.0009; Table 2).

**Table 2:** Univariate and multivariate analyses for disease-free survival

		Univariate analysis			Multivariate analysis		
			
Factors		HR	95% CI	*P*-value	HR	95% CI	*P*-value
All cases							
Tumor size	T3 vs. T1, T 2	2.67	1.32–4.92	0.0079	2.8	1.32–5.48	0.0082
LN meta.	Positive vs. negative	3.13	1.89–5.35	<0.0001	2.57	1.52–4.45	0.0004
Nuclear grade	3 vs. 1, 2	2.94	1.80–4.84	<0.0001	1.91	1.02–3.59	0.043
ER	Positive vs. negative	0.68	0.41–1.16	0.1545			
PR	Positive vs. negative	0.98	0.60–1.61	0.9443			
HER2	Positive vs. negative	2.25	1.30–3.76	0.0048	1.35	0.73–2.44	0.3261
Ki67	High vs. low	2.11	1.28–3.44	0.0036	1.12	0.61–2.04	0.7229
AR	High vs. low	0.41	0.25–0.68	0.0005	0.46	0.26–0.79	0.0052
ER-positive cases							
Tumor size	T3 vs. T1, T2	1.88	0.71–4.14	0.1863			
LN meta.	Positive vs. negative	3.29	1.76–6.45	0.0002	2.71	1.43–5.37	0.0021
Nuclear grade	3 vs. 1, 2	3.36	1.80–6.17	0.0002	2.37	1.25–4.43	0.009
PR	Positive vs. negative	1.60	0.80–3.57	0.1922			
HER2	Positive vs. negative	1.40	0.48–3.25	0.498			
Ki67	High vs. low	1.35	0.66–2.58	0.3915			
AR	High vs. low	0.30	0.16–0.54	0.0001	0.34	0.18–0.64	0.0009

HR, hazards ratio; CI, confidence interval; ER, estrogen receptor; PR, progesterone receptor; AR, androgen receptor; LN meta., lymph node metastasis; Ki67, cut-off 20%.

### Impact of the expression of the AR on the prognosis in ER-positive breast cancer by age

**Figure 3:**
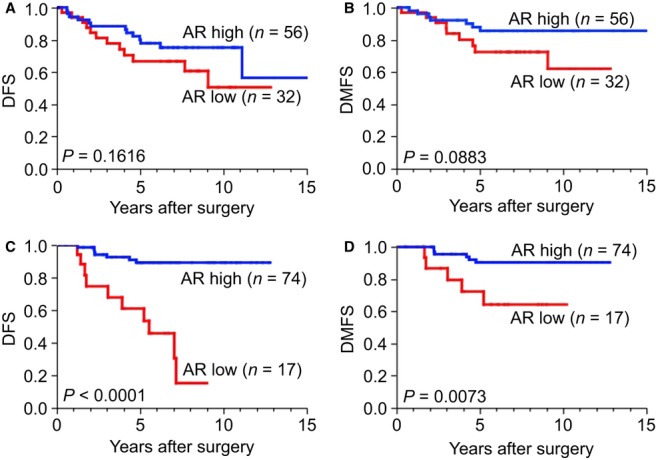
Impact of androgen receptor (AR) expression on the prognosis of patients with estrogen receptor (ER)-positive breast cancer by age. (A and B) In the younger (≤50-year-old) group, there was no significant association between AR expression and prognosis in terms of disease-free survival (DFS) (A) and distant metastasis-free survival (DMFS) (B). (C and D) On the other hand, in the older (≥51-year-old) group, a high AR expression level was significantly associated with a better prognosis in terms of both DFS (C) and DMFS (D).

The percentage of tumor cells with AR expression differed by age in ER-positive cases (Fig. 1E and F). Therefore, the association between AR expression and prognosis was evaluated by age in the ER-positive cohort. In patients ≤50 years old, there was no significant association between AR expression and DFS and DMFS (*P* = 0.1616 and 0.0883; Fig. 3A and B). On the other hand, in patients who were 51 or older, a high AR expression level was significantly associated with a better prognosis in terms of DFS and DMFS (*P* < 0.0001 and *P* = 0.0073; Fig. 3C and D). Therefore, AR expression was considered to have a profound effect on the prognosis of older (51 years or older) females with ER-positive breast cancer.

**Figure 4:**
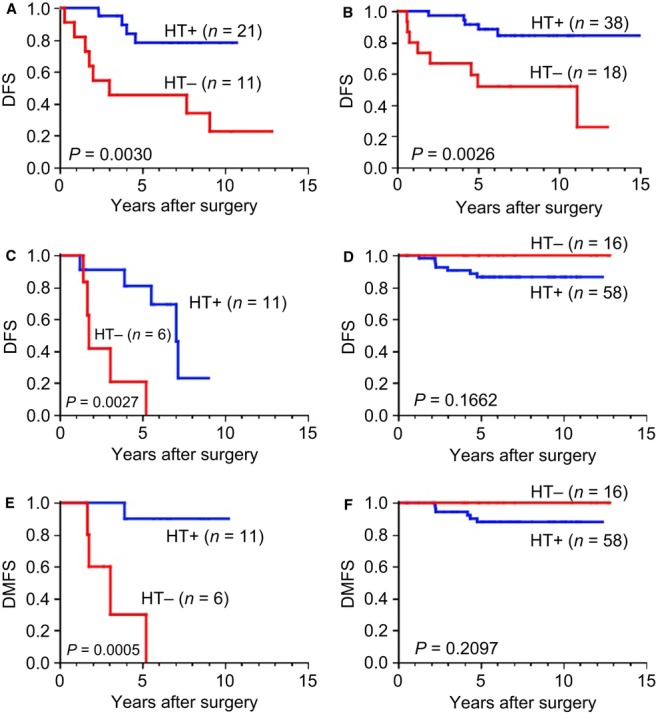
Association between administration of adjuvant hormone therapy and prognosis according to androgen receptor (AR) expression and age. (A and B) In the ≤50-year-old patients in both the AR-low (A) and AR-high (B) groups, disease-free survival (DFS) of the patients who received adjuvant hormone therapy was significantly better than that of the patients treated without adjuvant hormone therapy. (C and D) In patients who were 51 years old or older, the DFS of the patients treated with adjuvant hormone therapy was significantly better than that of the patients who did not receive adjuvant hormone therapy in the AR-low group (C), while there were no significant differences in the DFS of the patients with and without adjuvant hormone therapy in the AR-high group (D). (E and F) In the patients who were 51 years old or older, the distant metastasis-free survival (DMFS) of the patients treated with adjuvant hormone therapy was also significantly better than that of the patients who did not receive adjuvant hormone therapy in the AR-low group (E), while there were no significant differences in the DMFS of the patients with and without adjuvant hormone therapy in the AR-high group (F).

In addition, the association between the prescribed adjuvant hormone therapy and the prognosis according to AR expression and age was investigated in ER-positive patients. In the ≤50-year-old group, the DFS of the patients treated with adjuvant hormone therapy was significantly better than that of the patients without adjuvant hormone therapy in both the AR-low and AR-high groups (*P* = 0.0030 and 0.0026; Fig. 4A and B). On the other hand, in females who were 51 years old or older, the DFS of the patients treated with adjuvant hormone therapy was significantly better compared with the patients without adjuvant hormone therapy only in the AR-low group (*P* = 0.0027; Fig. 4C). On the other hand, in the AR-high group in these older female patients, there was no significant difference between the DFS of the patients with and without adjuvant hormone therapy (Fig. 4D). The DFS of the patients with low-AR expression was worse, in spite of the use of adjuvant hormone therapy, compared to the prognosis of patients with high-AR breast cancer (Fig. 4C). Notable events that affected survival were mainly local recurrence and contralateral breast cancer. In addition, in the older group, the DMFS of the patients treated with adjuvant hormone therapy was also significantly better compared with that in the patients without adjuvant hormone therapy, but only in the AR-low group (*P* = 0.0005; Fig. 4E), not in the AR-high group (Fig. 4F). In this older group, no significant difference was recognized in the tumor size, lymph node metastasis, nuclear grade, PR expression, HER2 status, or administration of adjuvant chemotherapy between the patients with and without adjuvant hormone therapy (data not shown). In terms of the kinds of hormone therapy, the prognosis of the patients treated with AIs was a little better than that of the patients treated with tamoxifen. However, the sample size was small and there were heterogeneities in the patients' backgrounds, so it is difficult to draw conclusions regarding the differences in the relationships between AR expression and response to AI or tamoxifen from our data.

### Associations between AR expression levels and biological phenotypes in ER-positive breast cancer patients by age

Because the association between AR expression and prognosis was different by age in the ER-positive cohort, the associations between AR expression levels and biological phenotypes were evaluated in the ER-positive cohort by age. In females who were 51 years old or older, high AR expression was associated with more nuclear grade 1 and less nuclear grade 3 disease (*P* = 0.0632), HER2 negativity (*P* = 0.0445), and a lower Ki67 index (*P* = 0.0015) (Table 3). However, there were no significant differences in the nuclear grade, PR, HER2, and Ki67 index between the AR-low and -high groups in patients ≤50 years old. These results suggest that, in females 51 years old or older, high AR expression was associated with less aggressive disease phenotypes (Table 3).

**Table 3:** Associations between androgen receptor (AR) expression and clinicopathological characteristics by age in the estrogen receptor (ER)-positive cohort

	≤50 (*n*=88)			>50 (*n*=91)		
		
	AR			AR		
				
Factors	Low (*n*=31)	High (*n*=57)	*P*-value	Low (*n*=17)	High (*n*=74)	*P*-value
Nuclear grade						
1	17 (54.8)	30 (52.6)	0.8442	5 (29.4)	42 (56.8)	0.0632
2	6 (19.4)	14 (24.6)		6 (35.3)	22 (29.7)	
3	8 (25.8)	13 (22.8)		6 (35.3)	10 (13.5)	
Progesterone receptor						
Negative	6 (18.8)	9 (15.8)	0.7219	8 (47.1)	30 (40.5)	0.6244
Positive	26 (81.2)	48 (84.2)		9 (52.9)	44 (49.5)	
HER2						
Negative	31 (96.9)	53 (93.0)	0.424	12 (70.6)	67 (90.5)	0.0445
Positive	1 (3.1)	4 (7.0)		5 (29.4)	7 (9.5)	
Ki67 index (%)	16.8±2.6	15.8±2.10	0.7534	18.9±2.1	11.3±1.0	0.0015

## Discussion

Previous studies reported that AR expression is positively correlated with ERα and PR expression, low-grade disease, and advanced differentiation [2]. Several recent studies revealed that the AR is an independent prognostic factor for the outcome of ERα-positive breast cancer [5, 7–10]. However, the relationship between the role of the AR and the menopausal status or age in breast cancer patients has not been reported. In this study, we confirmed that the expression of the AR is associated with the expression of other hormone receptors and less aggressive features, and that is an independent favorable prognostic factor in patients with ER-positive breast cancer. In addition, we showed that the expression of the AR increased by age in patients with ER-positive tumors, and also demonstrated that the association between AR-high expression and a good prognosis is observed in females who are 51 or older, but not significant in females who are 50 or younger. To the best of our knowledge, this is the first report that describes the difference in the expression of the AR and its impact on the prognosis of ER-positive breast cancer by age.

Peters and colleagues demonstrated that the AR potently inhibited the transactivational activity of ERα and the 17β-estradiol–stimulated growth of breast cancer cells [1]. The AR is able to bind to estrogen-responsive elements in ERα and prevent its growth-stimulatory effects, which is considered to be one of the mechanisms by which the AR is associated with a good prognosis in ER-positive breast cancer. Thus, the AR is considered to be a potential tumor suppressor for ER-positive breast cancer. The AR-mediated antiproliferative effects in breast cancer cells are influenced by the relative levels of endogenous AR and ERα [2]. Therefore, the failure to upregulate AR signaling may result in insufficient androgenic antagonism, thereby providing a growth advantage that contributes to disease progression in ER-positive breast cancer [2].

The balance between the stimulatory effects of estrogens and the inhibitory effects of androgens is a critical factor that regulates mammary cell proliferation in both normal and cancer tissues [21]. The mechanism underlying estrogen production dramatically changes before and after menopause. In premenopausal females, estradiol, which is the dominant type of circulating estrogen, is secreted mostly by the ovaries. In postmenopausal females, adipose tissue is the primary source of endogenous estrogen production, instead of the ovary. Androgens become an important source of estrogen through their aromatization to estradiol and estrone in the breast and other tissues in postmenopausal subjects [22]. After menopause, the circulating androgens are derived mainly from the adrenal gland [12]. Circulating estradiol levels decrease by 10-fold; however, the testosterone levels decrease by only 1.5-fold [13] after menopause. Moreover, the plasma androgen levels are much higher than those of estrogens in postmenopausal females [12]. Therefore, it is possible that the role of androgens is larger in postmenopausal than in premenopausal females with breast cancer. The mean age at natural menopause in Japanese females is 50 years [20]. Therefore, we divided all of our present cases into two groups based on age: ≤50 and ≥51 years old.

We found that AR expression was different by age only in ER-positive cases. AR expression was significantly higher in the subjects in the ≥51-year-old group, most of whom were likely postmenopausal. In addition, AR expression increased with age in ER-positive cases. We speculate that this is because circulating hormone levels affect the growth and proliferation of ER-positive breast cancer, and the circulating estrogen/androgen ratio decreases with age after menopause. This possibility should be confirmed in future studies.

We also demonstrated that the association between AR-high expression and a good prognosis is observed in females who are 51 or older, but that there were no significant associations in females who were 50 years old or younger. In females who were 51 years old or older, the high AR expression was associated with lower grade tumors, HER2 negativity, and a lower Ki67 index, which are all associated with less aggressive phenotypes. We also showed that there were associations between the use of adjuvant hormone therapy and DFS according to AR expression and age in ER-positive patients. In the ≤50-year-old patients in both the AR-low and -high groups, the DFS of the patients treated with adjuvant hormone therapy was significantly better compared to that of the patients without adjuvant hormone therapy (Fig. 4A and B). These results suggest that hormone therapy is effective and important in ER-positive premenopausal breast cancer patients, regardless of AR expression. On the other hand, in the older (51 and over) females in the AR-low group, the DFS and DMFS of the patients treated with adjuvant hormone therapy was significantly better than that of the patients without adjuvant hormone therapy (Fig. 4C and E), while there was no significant difference between the DFS and DMFS of the patients with and without adjuvant hormone therapy in the AR-high patients in this age group (Fig. 4D and F). These results indicate that AR-high expression is associated with a good prognosis regardless of the administration of adjuvant hormone therapy, while in the AR-low group, hormone therapy can improve the prognosis.

Most studies have come to the same conclusion that AR expression is related to a favorable prognosis in ER-positive breast cancer. The problem is that there has been high variability in the patient population, assay methods, and the analysis of the results of these previous studies. In terms of the methods used for IHC, large studies analyzed AR expression by IHC using TMA [9, 10], but this may have caused some bias due to the heterogeneity in each sample. We employed a whole section analysis, which is better than a TMA analysis, to evaluate AR expression in our study, resulting in less heterogeneity. In addition, the cut-off values for evaluating the positivity of AR expression in breast cancer have varied widely among studies: 1% [9, 10], low, AR < 10%; intermediate 10 ≤ AR < 50%; high, AR ≥ 50% [8], 10%; [7, 11], 75% [1]. In this study, we set the cut-off value for AR positivity as 75%, because of the similarity in the immune reactivity for AR to a previous report [1]. We also evaluated the outcomes of our patients using the median value, 83.3%. The results were almost the same; however, the *P*-value was smaller when using the cut-off value of 75%. When the cut-off value was set at 10%, no statistically significant difference in the DFS was observed between the AR-high and AR-low groups. When we divided the patients into three groups based on AR expression, with values of 0–10%, 10–75%, and 75%+, the ER positivity was 25%, 55.4%, and 84.5% (*P* < 0.0001), the PR positivity was 25%, 44.6%, and 61.9% (*P* = 0.0039), 83.3%, 45.2%, and 21.9% of tumors were nuclear grade 3 (*P* < 0.0001), and the Ki67 index was 43.0%, 20.9%, and 14.2%, respectively (*P* < 0.0001). There were no significant differences in tumor size and lymph node metastasis among these groups. These results suggest that the higher expression of the AR is associated with low-grade tumors. Therefore, an AR cut-off of 75% is considered to be suitable for our study. It is important to establish standard methods for detecting and evaluating the positivity of AR expression in the future.

There are emerging data regarding AR expression in breast cancers and the efficacy of hormone therapy, tamoxifen, and AIs. A preclinical study using an ER-positive breast cancer cell line showed that overexpression of the AR may cause resistance to tamoxifen [23]. If the expression of the AR can interfere with the activity of tamoxifen [23], the use of tamoxifen should be confined to AR-negative cancers. On the other hand, preclinical findings have suggested that AIs may be more effective in the presence of AR activated by androgens [12]. This suggests that AIs are better for AR-positive postmenopausal breast cancer patients. However, based on our data, the prognosis of postmenopausal females with ER-positive breast cancer is very good, even without the administration of adjuvant hormone therapy, if the expression of the AR is high. Further studies are necessary to explore these possibilities and confirm our present findings.

In conclusion, AR expression is associated with a less aggressive phenotype and a good prognosis in patients with ERα-positive breast cancer. This is considered to be a specific phenomenon for postmenopausal breast cancer patients. The evaluation of AR expression may therefore be useful to provide more adequate adjuvant therapy for postmenopausal females with ER-positive breast cancer.
